# Multiple Sinus Pauses in a Patient With COVID-19

**DOI:** 10.7759/cureus.14040

**Published:** 2021-03-22

**Authors:** Abdulbaril Olagunju, Beani Forst, Oleg Yakymovych, Beeletsega T Yeneneh

**Affiliations:** 1 Internal Medicine, Creighton University School of Medicine, Phoenix, USA; 2 Cardiovascular Diseases, Creighton University School of Medicine, Phoenix, USA

**Keywords:** coronavirus disease 2019, sinus pauses, hypoxemia

## Abstract

Cardiovascular complications in coronavirus disease 2019 (COVID-19) patients have been associated with poor prognosis. Myocarditis, acute coronary syndrome, heart failure, and arrhythmia have been reported.

We present a case of a 55-year-old female patient with no significant past medical history who was admitted due to COVID-19 induced acute hypoxemic respiratory failure. She developed multiple asymptomatic episodes of long sinus pauses as her oxygen requirements increased. These resolved without atropine and pacing as her respiratory status improved.

Hypoxemia, cytokine storm, dysautonomia, direct viral infiltration, and surrounding myocardial inflammation are thought to be responsible for bradyarrhythmias associated with COVID-19. Both symptomatic and asymptomatic cases have been reported. Hospitalized COVID-19 patients should be monitored closely on telemetry in order to promptly recognize any arrhythmia; hence preventing an unexplained rapid decline in cardiopulmonary status by intensifying care and managing the arrhythmia in a timely manner. Follow-up studies would be needed to determine the long-term outcomes of COVID-19 patients who developed bradyarrhythmias.

## Introduction

Coronavirus disease 2019 (COVID‐19) has been a major health crisis worldwide since its outbreak in Wuhan, China, with most clinically significant cases presenting initially as an acute decline in respiratory function [1]. A variety of cardiovascular manifestations have also been described, which unfortunately have been associated with poor prognosis [1]. These include myocarditis, acute coronary syndromes, heart failure, and arrhythmias [1-2]. Although tachyarrhythmias are the more common form of arrhythmia documented in patients with COVID‐19 [1], the incidence of bradyarrhythmias, including those due to sinus node dysfunction, is increasing [2-5]. We present a case of multiple asymptomatic sinus pauses in a patient with COVID-19.

## Case presentation

**Figure 1:**
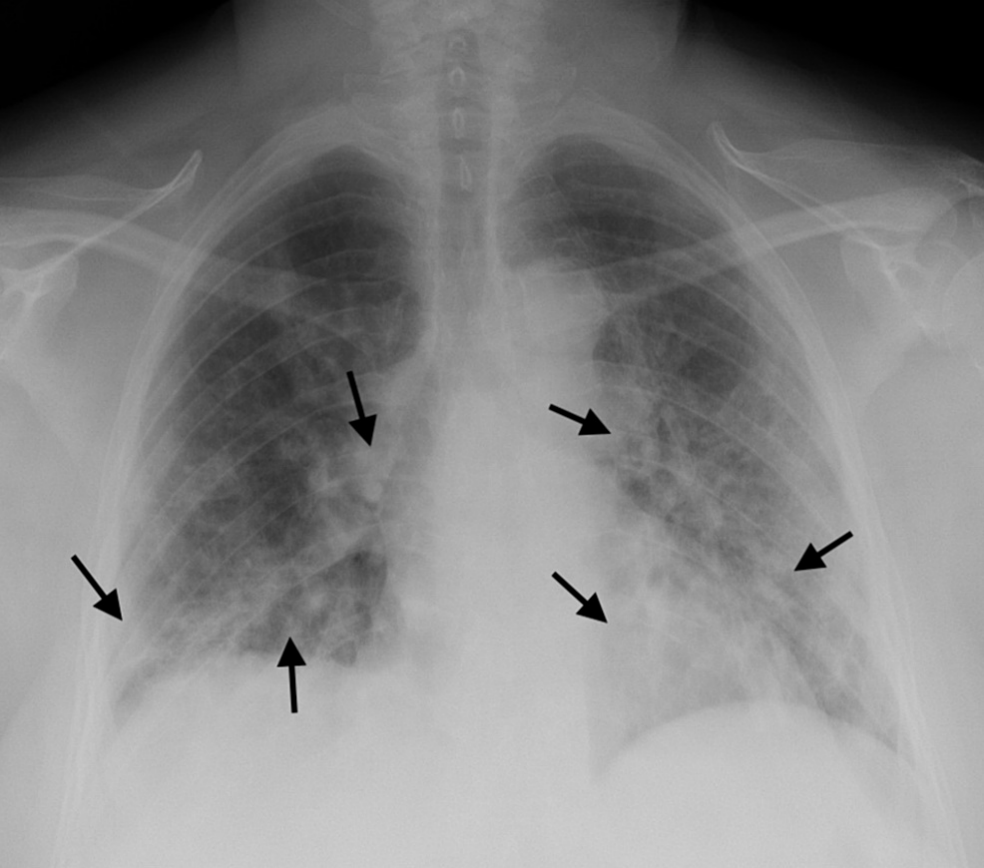
Chest radiograph showing bibasilar predominant multifocal opacities (arrows).

**Figure 2:**
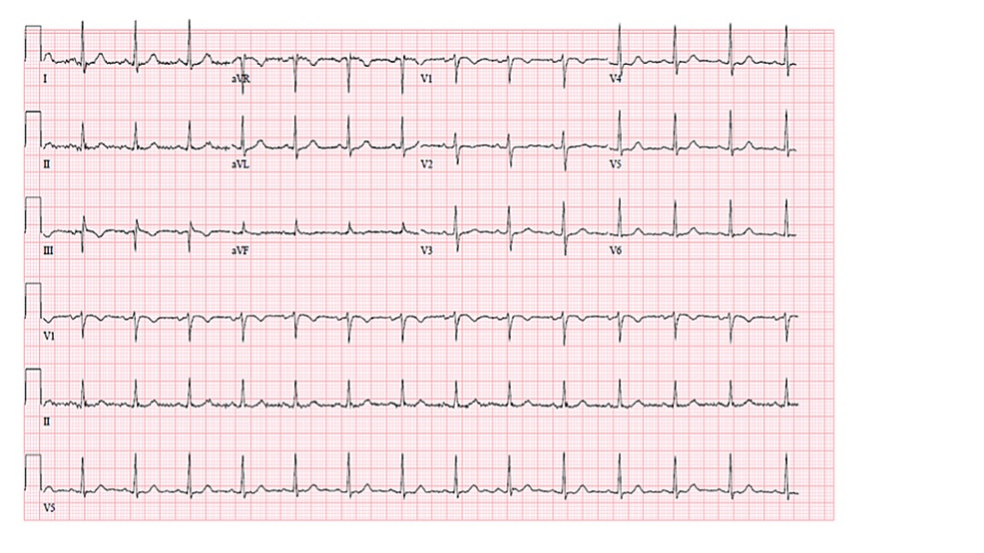
ECG on admission unremarkable for atrioventricular blocks. ECG, electrocardiogram

A 55‐year‐old morbidly obese female with no significant past medical history presented with a five‐day history of worsening dyspnea, fever, and chills. She and her family tested positive for COVID‐19 two days prior to her hospital admission and had been self‐isolating at home. On arrival to the ED, her oxygen saturation was 87% on room air, respiratory rate was 26 breaths per minute (b/m), and heart rate (HR) was 84 beats per minute (bpm). On physical examination, she was in respiratory distress with audible bibasilar rales on lung auscultation. She required 4 L of supplemental oxygen via a nasal canula to maintain oxygen saturation of 95%. Cardiovascular examination revealed regular rate and rhythm with normal S1 and S2 sounds; with no murmurs, rubs, gallops, jugular venous distention, or bilateral lower extremity edema. Her chest radiograph (Figure 1) was remarkable for the presence of bibasilar predominant multifocal opacities. Her body mass index (BMI) is 39.26 kg/m2. Electrocardiogram (ECG) (Figure 2) revealed sinus rhythm with normal axis, no ST‐T wave abnormalities, QTc prolongation or atrioventricular and bundle branch blocks. Nasopharyngeal swab polymerase chain reaction (PCR) confirmed COVID‐19 infection. Initial laboratory results were remarkable for leukocytosis (15.6 K/microL), elevated D‐dimer (281 ng/mL), procalcitonin (0.56 ng/mL), and C-reactive protein (CRP) (273.4 mg/L). She was admitted for supportive care and a 10‐day course of decadron. She declined remdesivir because it was an investigational drug at the time of her admission.

On day 1 of admission, she complained of occasional episodes of coughs with persistent dyspnea. Her oxygen was increased to 6 L on nasal canula as she became more hypoxic and tachypneic. Instead of having an expected physiologic tachycardia, her HR decreased but remained within normal average of 72 bpm. Lab result was remarkable for a downtrend in her leukocytosis (to 11.6 K/microL) and electrolytes remained normal. 

**Figure 3:**
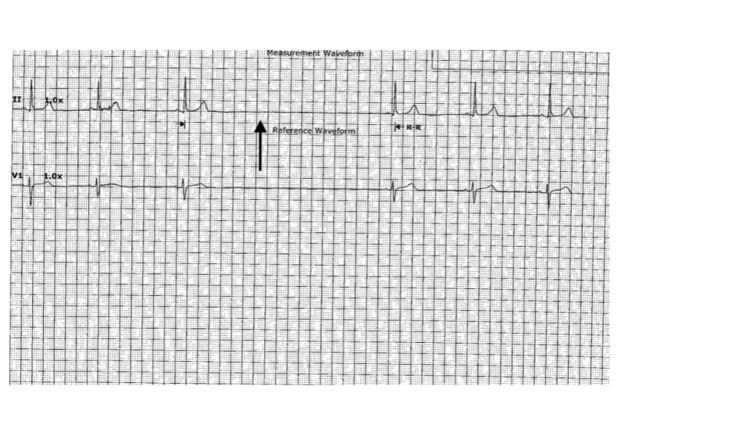
Sinus pause of 3.5 s (arrow) noted on telemetry while the patient was asleep at night.

**Figure 4:**
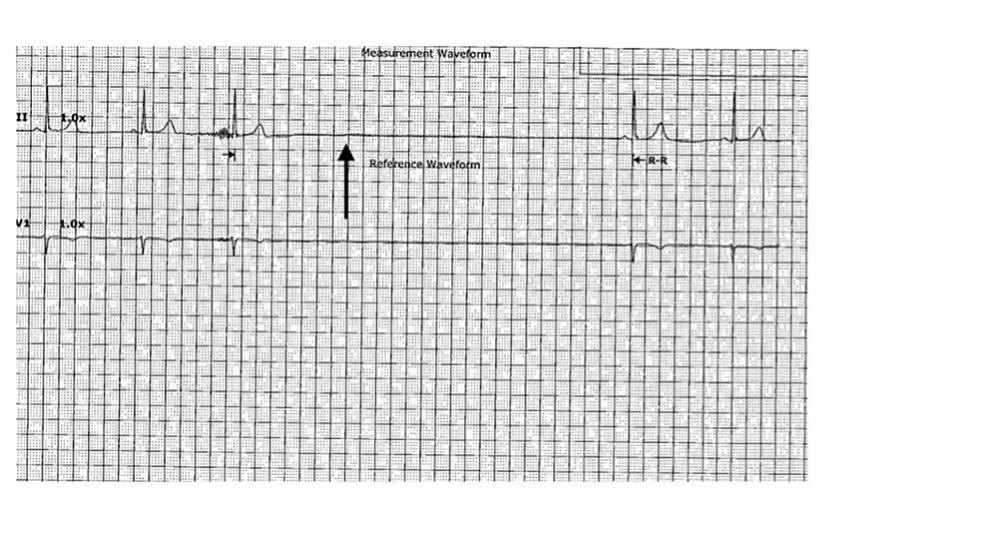
Longest sinus pause of over 5 s (arrow) noted on telemetry during the day while the patient was awake.

On day 2 of her admission, her dyspnea persisted, and her oxygen needs increased to 10 L on a high-flow bubbler. Her HR continued to decrease and she became bradycardic (55 bpm). In addition to this, multiple overnight episodes of sinus pause ranging from 3.5 to 5 s were evident on the telemetry monitor while she was asleep (Figure 3). Upon awakening on day 3, she was persistently bradycardic with heart rate in the 40 s. Surprisingly, she also continued to have episodes of sinus pauses throughout the day with the longest being over 5 s (Figure 4). Bedside assessments during these episodes were unremarkable for worsening of her dyspnea, chest pain, near syncope or syncope, confusion, lethargy, or other altered mental status.

**Figure 5:**
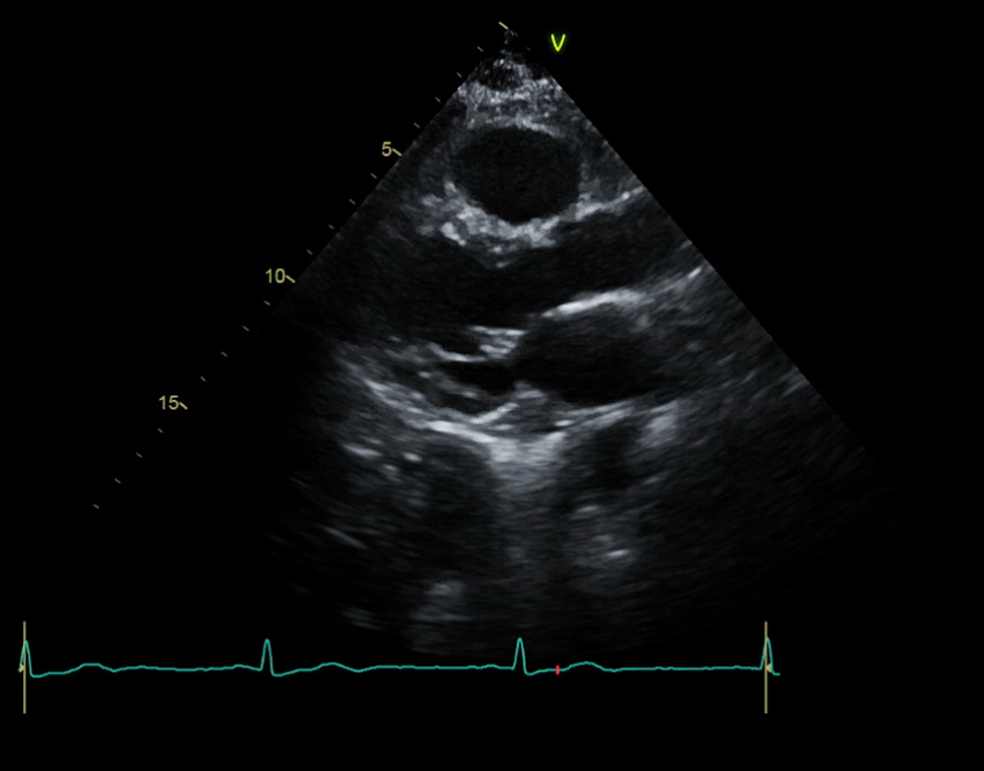
Parasternal long axis view of transthoracic echocardiogram unrevealing for structural abnormalities.

A transthoracic echocardiogram was unremarkable for structural abnormality with an ejection fraction of 66% (Figure 5). She had not received any sinoatrial or atrioventricular node blocking or QTc prolonging medications during this admission and denied taking any over-the-counter medications at home. No electrolyte or thyroid abnormalities were present on her labs. Her STOP-Bang score was 2 meaning she had a low risk for obstructive sleep apnea (OSA). Due to her absence of symptoms, decision was made against placing a permanent maker unless needed. The plan was to have atropine and transcutaneous pacer at bedside just in case she developed symptoms.

**Figure 6:**
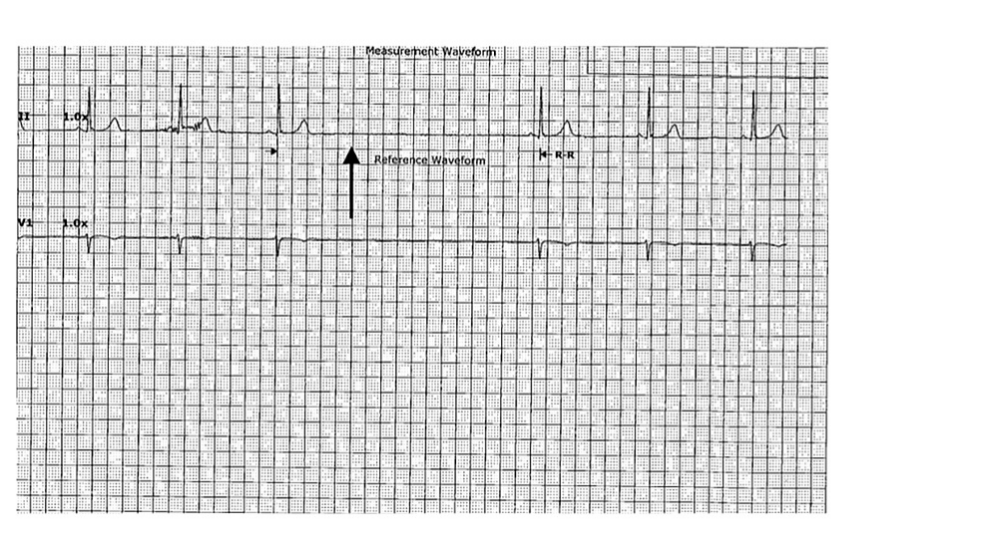
Sinus pause duration decreased to 3.5 s (arrow) while patient was asleep.

**Figure 7:**
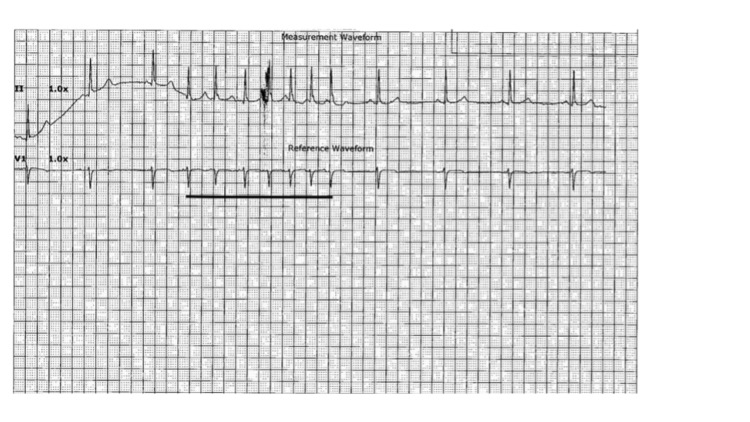
Telemetry strip on day 6 revealing sinus bradycardia at a rate of 50 bpm with a 2.5 s run of an ectopic atrial rhythm (span of solid black line); no sinus pause was noted.

Starting from day 4, the frequency and duration of sinus pause episodes started decreasing. These coincided with her dyspnea improving and her oxygen requirements also decreasing. She experienced a single episode of 3.5 s sinus pause on day 5 of admission while she was asleep (Figure 6). Starting from day 6, no episodes of sinus pauses were recorded on telemetry (Figure 7). Her HR gradually increased from the 40 s to normal at 77 bpm. She was discharged home on day 8 with 2 L of oxygen. 

## Discussion

This patient’s sinus pauses in the absence of known causes such as OSA, nodal blocking medications, structural heart disease, history of myocardial infarction, hypothyroidism, and electrolyte abnormalities [3] make COVID‐19 infection a possible cause. The increase in the episodes of sinus pauses with an increase in oxygen requirement, and the decrease in the frequency of the episodes as her oxygen requirement improved also supports the idea of COVID‐19 as a likely cause. The exact mechanism by which COVID‐19 causes this is not known. Speculations such as direct viral infiltration of the cardiac conduction system [2-3], surrounding myocardial inflammation [2], hypoxemia, impact of circulating cytokines [2, 4], and infiltration of the cardiorespiratory brainstem centers resulting in dysautonomia [5] have been put forward. Of these, the latter three might explain the sinus node dysfunction (SND) in this patient. The absence of elevated troponin, atrioventricular node, and bundle branch conduction abnormalities on ECG makes direct viral infiltration and myocardial inflammation less plausible mechanisms. Infiltration of the cardiopulmonary brainstem is thought to be via a transsynaptic viral transport through afferent chemoreceptors and mechanoreceptors from infected lungs [5]. Circulating cytokines have been associated with suppression of cardiac conduction [2, 6]. Hence, because cytokine/inflammatory storm correlates with the need for supplemental oxygen [7-8], the reduction in the frequency of sinus pauses which correlated with the decreased oxygen need might be explained by an improvement in systemic inflammation. The absence of symptoms in this patient precluded the placement of a permanent pacemaker. The prognostic impact of sinus pauses in COVID‐19 infection is not well known due to the relative paucity of reported cases. While asymptomatic cases that have been described in the literature till date seem to have recovered from the infection [3-4], most of the reported cases with symptomatic sinus node dysfunction associated with COVID‐19 have had poor prognosis despite permanent pacemaker implantation [9]. This could indicate the need for aggressive treatment and intensive monitoring. In addition, the association of sinus pauses with COVID‐19 means hospitalized patients might benefit from telemetry monitoring. This will detect new‐onset conduction abnormalities and could prevent an unexplained sudden deterioration in cardiopulmonary function. Whether a history of asymptomatic sinus pauses due to COVID‐19 would increase the risk of symptomatic recurrence in future is yet to be known and follow‐up of these patients might be needed to determine such.

## Conclusions

Hypoxemia, direct viral infiltration of the cardiac conduction system, cytokine storm, myocardial inflammation, and autonomic dysfunction are thought to be responsible for COVID-19 associated sinus node dysfunction. Both symptomatic and asymptomatic cases have been reported. Permanent pacemakers have been used in symptomatic patients while asymptomatic patients have been closely monitored until resolution. The resolution of sinus pauses in our patient seemed to correlate with improvement in respiratory status. Telemetry monitoring might be invaluable in detecting sinus pauses in hospitalized COVID‐19 patients. Long‐term follow‐up might be needed to determine the risk of recurrence in these patients.
